# Roles and mechanisms of circular RNA in respiratory system cancers

**DOI:** 10.3389/fonc.2024.1430051

**Published:** 2024-07-15

**Authors:** Nan Yang, Mengwen Jiao, Yuewen Zhang, Shaokang Mo, Ling Wang, Jianqing Liang

**Affiliations:** ^1^ School of Basic Medical, Gansu University of Chinese Medicine, Lanzhou, China; ^2^ School of Public Health, Gansu University of Chinese Medicine, Lanzhou, China; ^3^ Department of Obstetrics and Gynecology, The 940th Hospital of Joint Logistics Support Force of Chinese People’s Liberation Army, Lanzhou, China

**Keywords:** circRNA, respiratory system, non-small cell lung cancer, nasopharyngeal cancer, Laryngeal squamous cell carcinoma

## Abstract

Circular RNAs (circRNAs) constitute a class of endogenous non-coding RNAs (ncRNAs) that lack a 5’-ended cap and 3’-ended poly (A) tail and form a closed ring structure with covalent bonds. Due to its special structure, circRNA is resistant to Exonuclease R (RNaseR), making its distribution in the cytoplasm quite rich. Advanced high-throughput sequencing and bioinformatics methods have revealed that circRNA is highly conserved, stable, and disease- and tissue-specific. Furthermore, increasing research has confirmed that circRNA, as a driver or suppressor, regulates cancer onset and progression by modulating a series of pathophysiological mechanisms. As a result, circRNA has emerged as a clinical biomarker and therapeutic intervention target. This article reviews the biological functions and regulatory mechanisms of circRNA in the context of respiratory cancer onset and progression.

## 1 Introduction

Circular RNA (circRNA), a novel kind of non-coding RNA (ncRNA) created through the reverse splicing of precursor mRNA (pre-mRNA), lacks a 5’cap and a 3’ poly (A) tail and is converted into a closed circular molecule via covalent bonding (1, 2). This covalent closed loop structure, unlike the majority of linear messenger RNAs (mRNAs) and long non-coding RNAs (lncRNAs), is not impacted by RNA exonucleases and not readily disintegrated, making it a highly promising biomarker (3, 4).

**Figure 1:**
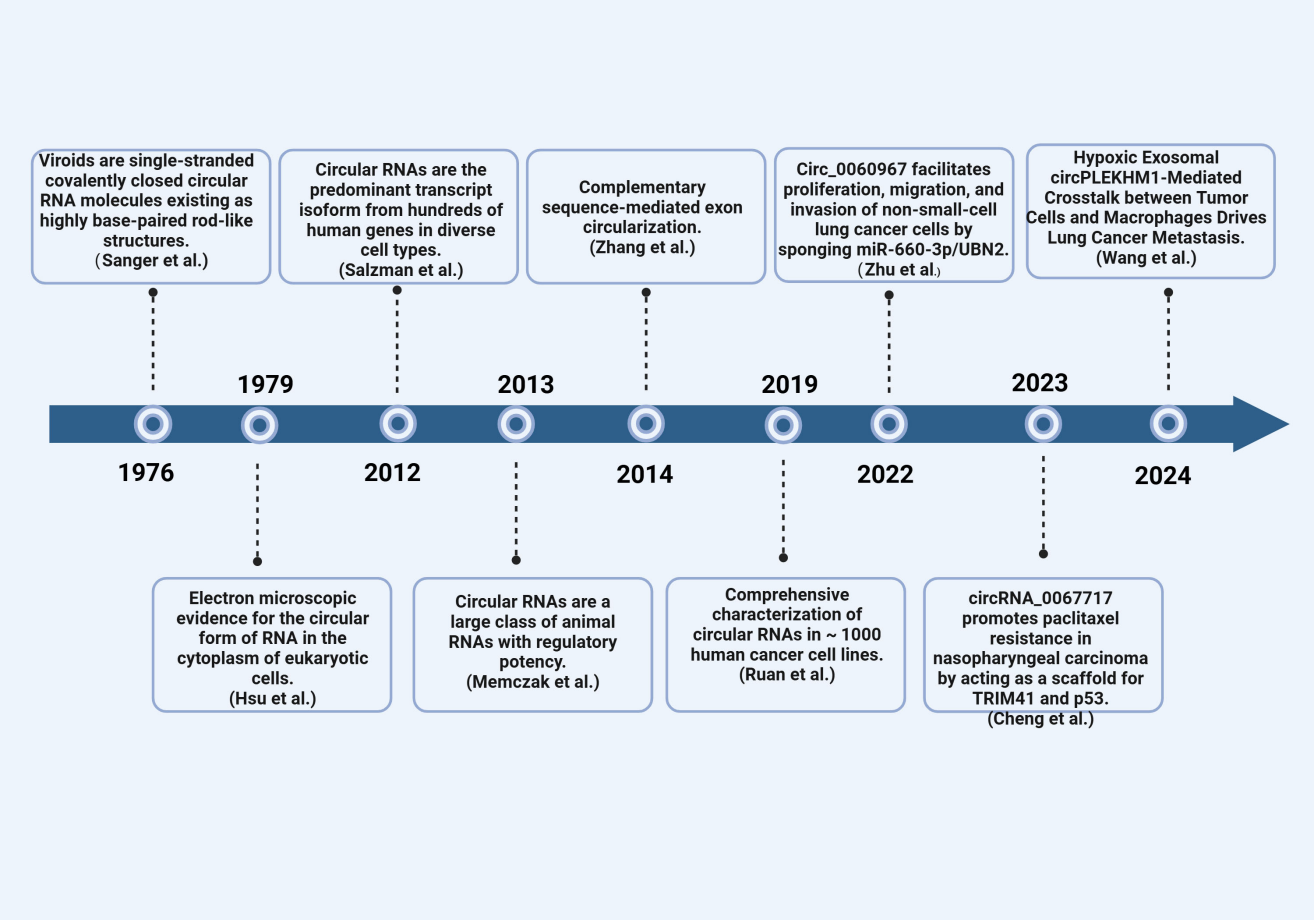
The discovery of circRNAs. Timeline of important events related to the discovery, research, and development of circRNA.

Single-stranded, thermally stable circRNA structures were first observed in 1976 by Sanger et al. (German scientists) under an electron microscope in a process that involved denaturing plant RNA viruses (5). American scientists later observed circRNA in eukaryotic (Hela) cytoplasm using an electron microscope in 1979 (5). Initially, circRNA was considered a “transcription artifact or splicing noise” and was not explored due to its low transcription abundance and technological limitations (6, 7). It was until 2012 that Salzman et al. discovered a large number of circRNAs in the human body through transcriptome sequencing of bone marrow samples from leukemia patients using high-throughput sequencing technology (8). Since then, multiple studies have confirmed the widespread existence of circRNA in various human tissues. For example, several studies have identified and characterized circRNA in various systemic cancers (9–11). Moreover, some studies have demonstrated a strong correlation between circRNA and the onset and progression of various malignant tumors of the respiratory system, including Nasopharyngeal Carcinoma (NPC) (12, 13), Laryngeal Squamous Cell Carcinoma (LSCC) (14), and Non-small Cell Lung Cancer (NSCLC) (15). Consequently, circRNA has emerged as a promising novel target for detecting and managing respiratory illnesses, as well as a significant molecular regulator in respiratory cancers. This article summarizes the biological activities and regulatory roles of circRNA in the respiratory system, aiming to inform the clinical diagnosis and treatment of respiratory cancers (**Figure 1**).

## 2 The biogenesis and formation mechanism of circRNA

As earlier stated, circRNA is synthesized via reverse splicing. During biogenesis, circRNA’s downstream 5’splice site is connected to the upstream 3’ splice site of the same or another exon, forming a covalently closed circular transcript and a linear transcript with selective splicing to skip the exon at the reverse splice junction (14). In other words, circRNA synthesis involves the typical spliceosome mechanism, where circRNA transcription competes with typical pre-mRNA splicing and impacts the expression of typical genes (15).

**Figure 2:**
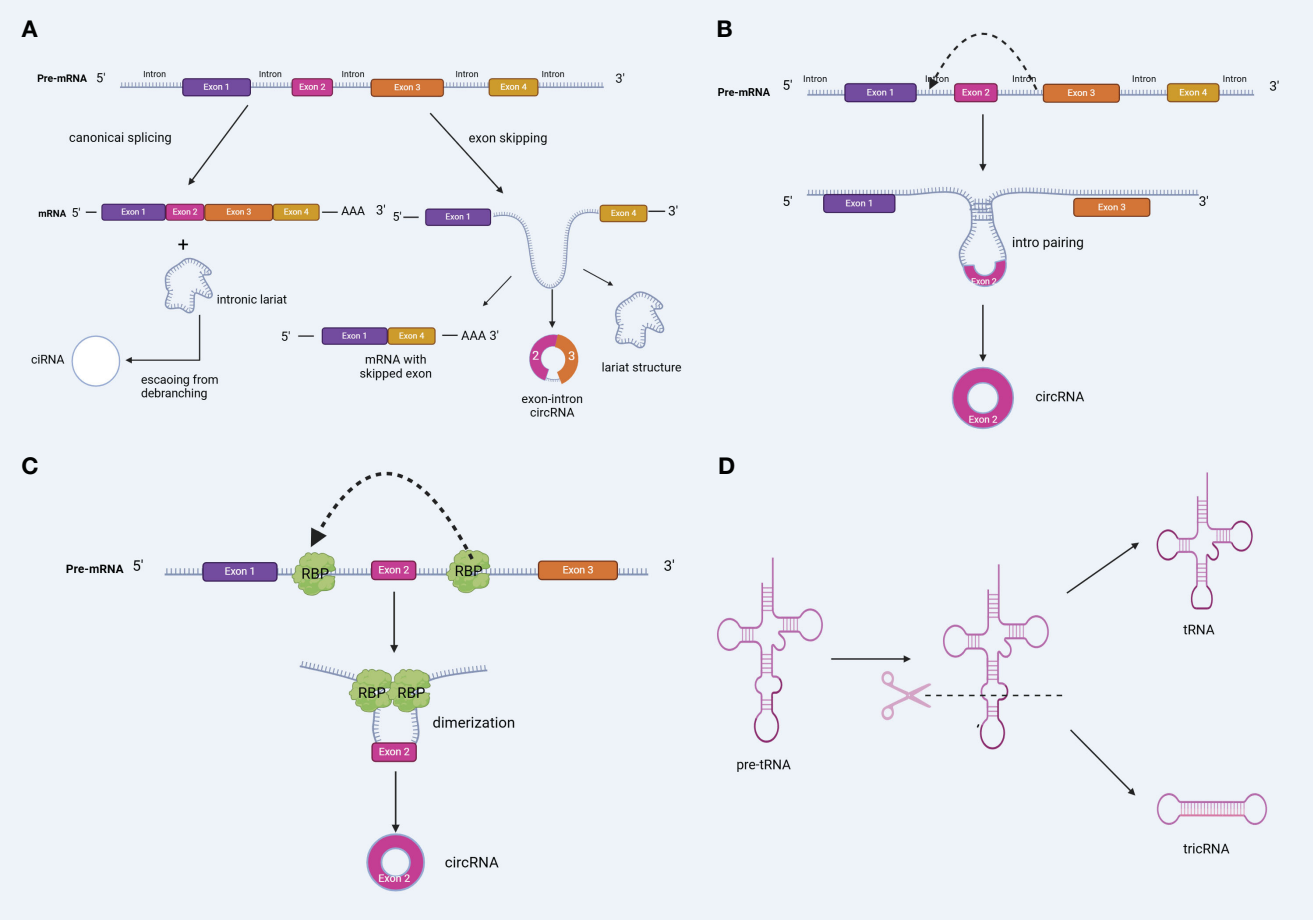
Biogenesis of circRNA **(A)** lariat-driven circularization (exon skipping) **(B)** Back-splicing can be induced by intron pairing (intron pairing-driven cyclization). **(C)** RNA-Binding Protein (RBP)-mediated cyclization,. **(D)** tricRNA biogenesis.

Based on their origin, circRNA formation patterns can be classified into four categories: exon-derived circRNAs (ecRNAs), intron-derived circRNAs (ciRNAs), exon-intron circRNAs (EIciRNAs; comprising both exons and introns), and intergenic circRNAs (16, 17). Notably, ecRNAs contain only reverse spliced exons and are mainly found in the cytoplasm. They are formed by turning off, jumping, and removing introns, and are the most common types of circRNAs (18). On the other hand, intron-only and exon- and intron-derived circRNAs are mainly located in the nucleus (19). Notably, different types of circRNAs are produced via different mechanisms. Presently, circRNA biogenesis is generally believed to involve four different mechanisms: LARAT-driven cyclization (exon jumping), direct reverse splicing (intron pairing-driven cyclization), RNA-Binding Protein (RBP)-mediated cyclization, and tricRNA biogenesis (intron splicing-driven cyclization) (20–22) (**Figure 2**).

## 3 Biological functions of circRNA

**Figure 3:**
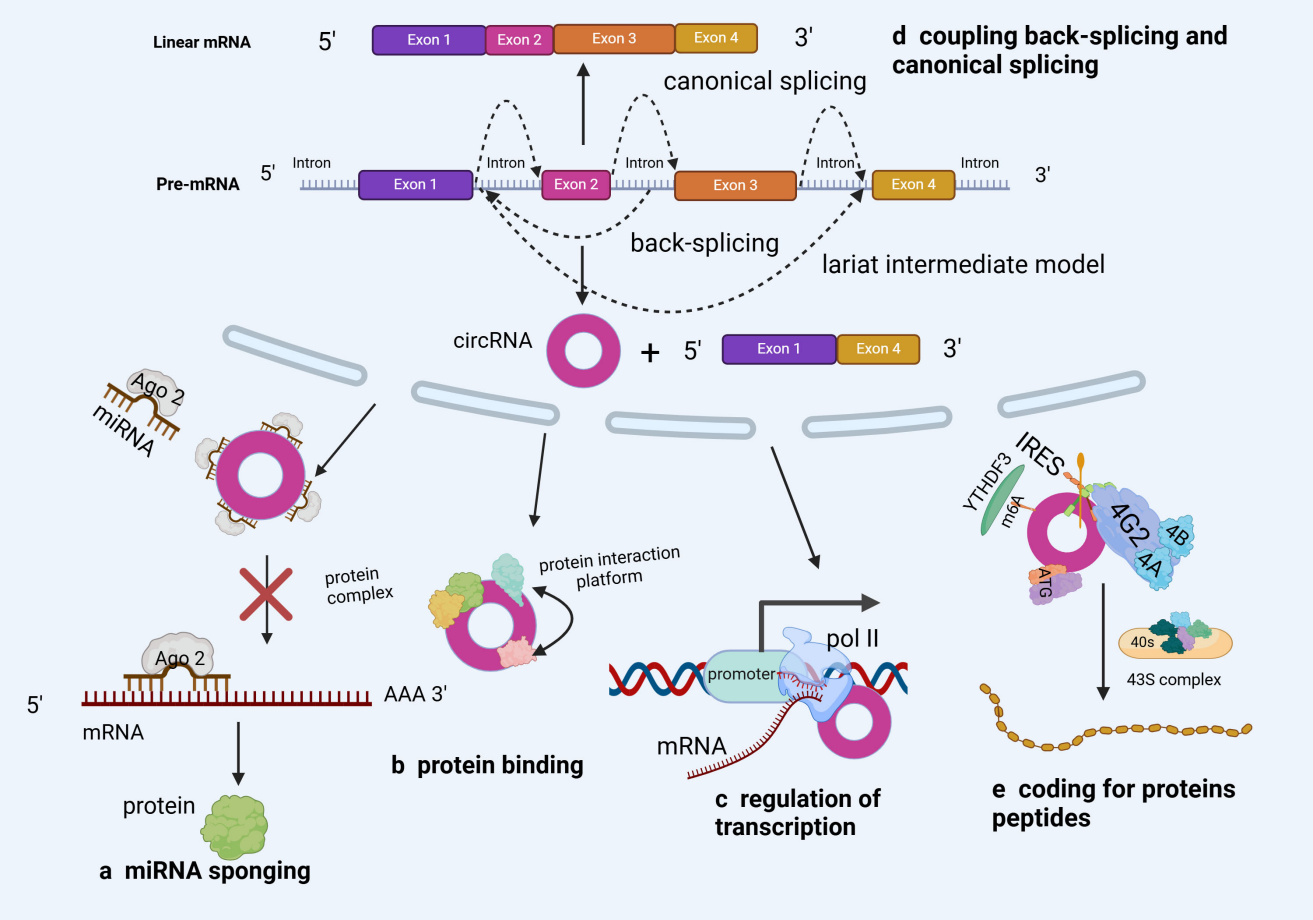
Biological functions of circRNA **(A)** The most common function of circRNAs is miRNA sponging. By sponging miRNAs, circRNAs inhibit miRNA capacity to perform their post-transcriptional inhibition. **(B)** circRNAs can act as RBP decoys, protein sponges or scaffolds to regulate target gene expression. **(C)** circRNAs can regulate parental genes in response to RNA polymerase II. **(D)** circRNA backsplicing can selectively compete with precursor mRNA typical splicing and affect typical gene expression rates. **(E)** Some circRNAs have coding potential and are translated into proteins.

Although its biological function remains debatable (23), circRNA is widely present in eukaryotes and could function as a miRNA “sponge” (24), protein “sponge” or “scaffold” (25), and translation template (26, 27). However, compared to linear mRNAs from the same gene, most circRNAs are expressed at lower levels (28). This suggests that the majority of circRNAs might be functionally irrelevant and merely inconsequential byproducts of alternative splicing (29, 30). Therefore, although circRNAs have existed for long, research on their functions is still at infancy (31, 32). Nonetheless, most research results have shown that circRNAs have biological functions (**Figure 3**).

### 3.1 Binding miRNAs as ceRNAs

The primary function of circRNAs is to bind to miRNAs as a ceRNA and block their biological activities, thus regulating the expression of target genes (33). Wu et al. (34) reported a substantial circWHSC1 expression in NSCLC cells, implying that it could be a useful biomarker for NSCLC diagnosis and prognosis (**Figure 3A**). Based on its functional mechanism, circWHSC1 could interact directly with miR-590–5p, upregulating SOX5 (a carcinogenic molecule) and accelerating malignant NSCLC development. Furthermore, some studies reported that circIFI6 may facilitate Esophageal Squamous Cell Carcinoma (ESCC) progression. Additionally, CircIFI6 regulates the expression of hub genes (HK2, MYLK, CDKN1A, CDH3, and TGFBI) by adsorbing miR-497–5p and miR-195–5p via the ceRNA mechanism, regulating the course and prognosis of esophageal cancer (35). These findings demonstrate the regulatory role of circRNAs (as ceRNAs) in tumor development, offering novel insights into circRNA-related cancer research.

### 3.2 Interaction with RBP

Notably, RBPs constitute a large class of proteins involved in the regulation of transcript splicing, transport, modification, stability, and translation (34, 36) (**Figure 3B**). A growing body of research has shown the significant therapeutic potential of circRNA-RBP interaction, as well as its critical involvement in the onset and progression of many cancers (37–39). For example, through luciferase and RNA pull-down assays, Tang et al. (40) confirmed that circ-0000009 suppresses Lung Adenocarcinoma (LUAD) development by upregulating PDZD2, a tumor suppressor, in a ceRNA- and RBP-dependent manner. This finding opens up new avenues for LUAD diagnosis and treatment. Moreover, the interaction of the Human antigen R (HuR) protein and circAGO2 enhanced the latter’s competitive enrichment in the 3’Untranslated Region (UTR) of the target gene. Consequently, AGO2 binding decreased, AGO2 miRNA-mediated gene silencing was suppressed, and tumor formation and invasion increased (41). In summary, circRNA’s protein sponge activity opens up new avenues for cancer prognosis monitoring, treatment, and early diagnosis.

### 3.3 Regulating parent gene transcription

It has been reported that circRNAs may regulate parental genes through the action of RNA polymerase II (**Figure 3C**). On the one hand, circRNA can form R-loops with its production sites, impacting DNA replication, repair, and transcription. On the other hand, circRNA could co-activate with Transcription Factors (TF) to regulate transcription (42). Ribonuclease H1 (RNase H1) is an endonuclease that degrades RNA strands in the R loop and may also degrade some ciRNAs. In a previous study, RNase H1-mediated degradation of the transcriptional R loop by cinkrd52 enhanced transcriptional elongation. Specifically, ciRNA was eliminated through RNase H1, thus facilitating transcriptional elongation (43). Furthermore (44), and STAT-3 are representative TFs that have long been associated with Cancer Stem Cells (CSCs) (45), and the absorption of miR-29a/b/c-3p by circ-0043800 in hepatoblastoma cells could induce STAT-3, thus promoting tumor growth (46).

### 3.4 Adjustable pre-mRNA splicing

Most anti-splicing events are generated by exons with typical splicing sites that co-express with homologous linear mRNA at their host gene sites (**Figure 3D**). In this regard, circRNA anti-splicing competes with pre-mRNA selective splicing, potentially downregulating linear mRNA (45, 47). According to research, circRNA regulates selective splicing events, which are quite common in cancer (48). Additionally, Ma et al. (49) discovered that circZFR enhances Oxidative Phosphorylation (OXPHOS) in LUAD via selective splicing regulation. Specifically, CircZFR activated the AKT mTOR pathway by stabilizing Heterogeneous Nuclear Ribonucleoprotein l (HNRNPL1), thereby regulating the selective splicing of MYO1B full-length transcript (MYO1B fl) and promoting OXPHOS and cell proliferation. These findings indicate that circZFR modulates OXPHOS to exert carcinogenic effects via selective splicing regulation, offering novel insights into cancer research.

### 3.5 Translation

Open Reading Frames (ORFs) in certain circRNAs have been established to encode functional proteins or peptides (**Figure 3E**). The m6A-mediated cap-independent translation start and the Internal Ribosome Entry Site (IRES) are the two putative pathways for circRNA translation (50–52). Wang et al. (53) confirmed that circSEMA4B inhibits breast cancer progression by encoding a new protein, SEMA4B-211aa, thus regulating AKT phosphorylation. Specifically, SEMA4B-211aa inhibits AKT phosphorylation by competing with p110 to bind to p85. Additionally, Zheng et al. (50) discovered that circYthdc2 has an m6A- and IRES sites-driven ORF and encodes a functional peptide comprising 170 amino acids (Ythdc2–170aa). By specifically targeting the Stimulator for Interferon Genes (STING), Ythdc2–170aa can suppress the STING-mediated antiviral immune response while promoting the replication of RNA viruses.

## 4 Roles and mechanisms of circRNA in respiratory cancers

Besides its involvement in various physiological and autoimmune diseases, circRNA also plays an important role in the onset and progression of various malignant tumors (54), cell proliferation (55), cell apoptosis, and metastasis (56). With advancements in bioinformatics technology, an increasing number of circRNAs have been shown to play different biological functions in various cancer systems via multiple pathways (57, 58). Some of these biological functions include the proliferation and differentiation of various digestive system malignancies, including Hepatocellular Carcinoma (HCC) (59–61), Colorectal Cancer (CRC) (62–64), and Gastric Cancer (GC) (65–67), among others. Additionally, circRNA is involved in the onset and progression of various respiratory cancers, including Laryngeal Squamous Cell Carcinoma (LSCC) (68), Nasopharyngeal Carcinoma (NPC) (69), and NSCLC (70–72),, among others. Finally, circRNA has also been reported to participate in the proliferation and migration processes of various reproductive system malignancies, including ovarian cancer (73–75), cervical cancer (76–78), and prostate cancer (79, 80). Therefore, it is plausible that circRNA has emerged as a novel and unique epigenetic regulatory factor (81). This review focused on molecular mechanisms of circRNA regulation in respiratory cancers, which is summarized in the subsequent sections.

### 4.1 Non-small cell lung cancer

**Figure 4:**
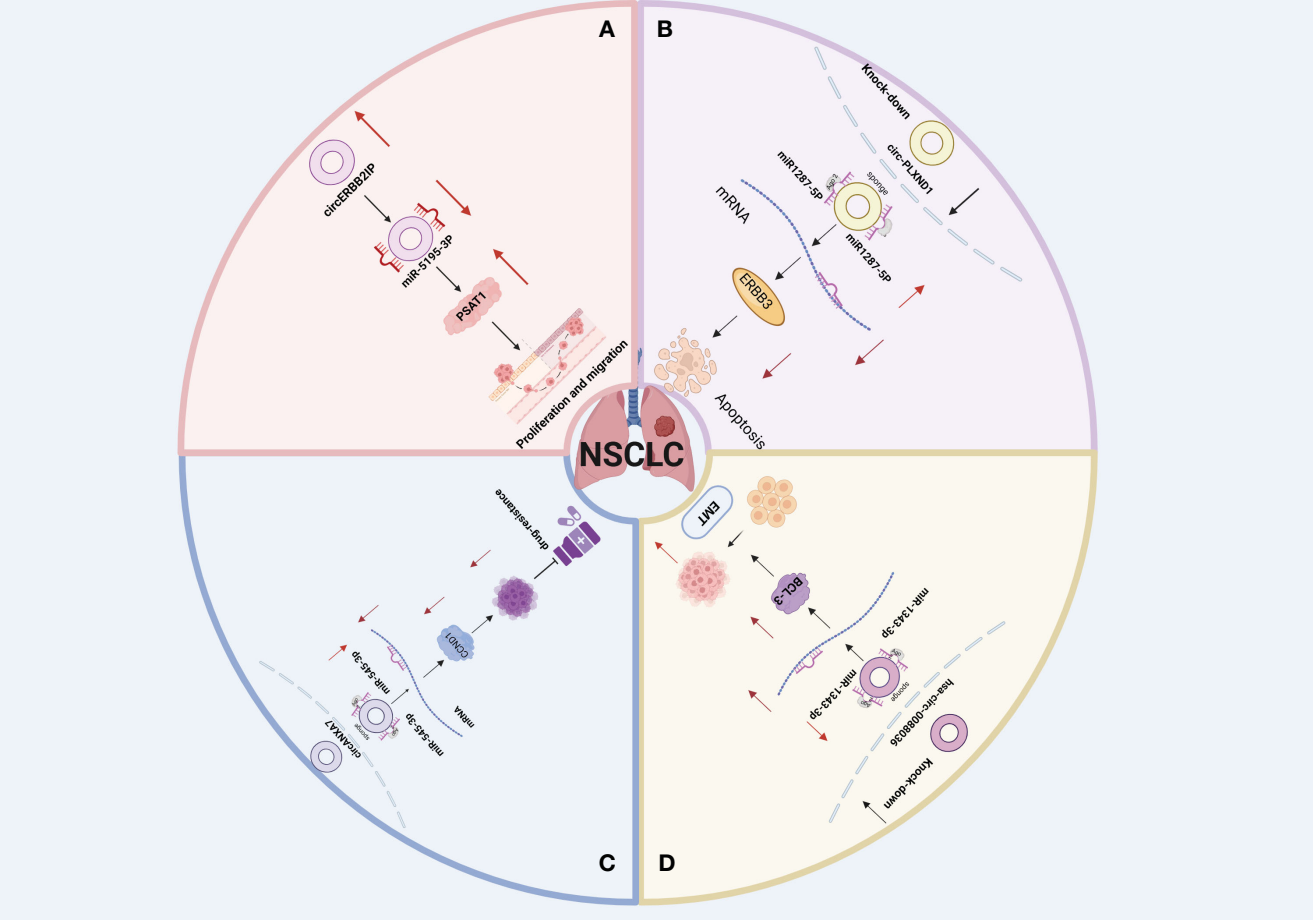
**(A)** Schematic illustration of the circERBB2IP/miR-5195–3p/PSAT1 axis mediating non-small cell lung cancer (NSCLC) progression. **(B)** Downregulation of circ_PLXND1 inhibits tumorigenesis of non-small cell lung carcinoma via miR-1287–5p/ERBB3 axis4c. **(C)** Hsa_circ_0088036 promotes non-small cell lung cancer progression by regulating miR‐1343‐3p/Bcl‐3 axis through EMT signaling. **(D)** circANKRD28 inhibits cisplatin resistance in non-small-cell lung cancer through the miR-221–3p/SOCS3 axis.

Non-Small Cell Lung Cancer (NSCLC) is a common malignant tumor that originates from the bronchial mucosa or gland (82). NSCLC is the most common histological subtype of lung cancer, constituting about 85% of cases (82). The two primary subtypes of NSCLC are Lung Squamous Cell Carcinoma (LUSC) and Lung Adenocarcinoma (LUAD), which together account for approximately 90% of all cases (83–85). Lung Large Cell Carcinoma (LLCC) makes up about 10% of lung cancers (86). CircRNA, acting as either a driving or inhibitory factor, regulates the occurrence and progression of NSCLC through various pathological and physiological mechanisms. These include Epithelial-Mesenchymal Transition (EMT), cell proliferation, metabolism, apoptosis, drug resistance, and migration (70, 87, 88) (**Figure 4**).

#### 4.1.1 Proliferation and migration

Forkhead box M1 (FOXM1) is a member of the conserved forkhead box (FOX) transcription factor family, recognized for its role as a multifunctional oncoprotein regulating the expression of numerous cancer-promoting genes (89). In a study by Cheng et al. (90), analysis of LUSC patient specimens revealed that circTP63 was positively correlated with tumor size and TNM stage. High circTP63 expression influenced the cell cycle progression and proliferation of LUSC cells both *in vitro* and *in vivo*. Mechanistically, circTP63 functioned as a competing endogenous RNA (ceRNA), binding to miR-873–3p and neutralizing its suppressive effect on the target gene FOXM1. This elevation of FOXM1 subsequently enhanced the expression of downstream cell cycle-related genes CENPA and CENPB, driving cell cycle progression and cell proliferation. Moreover, Facin Actin-bundling Protein 1 (FSCN1) is an important protein that regulates cancer cell migration and invasion (91). Zhang et al. (92) explored the function and mechanism of circSATB2 in NSCLC and confirmed through dual luciferase and quantitative Polymerase Chain Reaction (qPCR) assays that circSATB2 can directly bind to miR-326 to regulate FSCN1 expression and promote NSCLC progression. Additionally, Peng et al. (93) conducted animal experiments and discovered that knocking down circERBB2IP inhibited NSCLC proliferation and migration (**Figure 4A**). Furthermore, previous *in vivo* experiments implied some correlation between circERBB2IP and TNM grading, Lymph Node Metastasis (LNM), and tumor size in NSCLC patients. Additionally, functional analyses revealed that circERBB2IP could drive NSCLC growth by binding to miR-5195–3p to positively regulate Phosphoserine Aminotransferase 1 (PSAT1) expression. These findings highlight circRNA as a potential biomarker and therapeutic target for clinical NSCLC detection and treatment.

#### 4.1.2 Apoptosis

Accumulating evidence suggests that (94) tumor cell apoptosis mechanisms (94). The bromodomain PHD finger transcription factor (BPTF), a core subunit of nucleosome-remodeling factor (NURF) complex, serves as a transcriptional regulator which facilitates tumor progression (95). BIRC6, as a member of the inhibitors of apoptosis protein (IAP) family, can suppress the expression of the BPTF target gene c-MYC and enhance LUAD progression by promoting apoptosis and cell cycle blockage. A study by Chen et al. (96) demonstrated that circ-100549 serves as a ceRNA by sponging miR-95–5p to upregulate BPTF expression, thus upregulating BIRC6 expression at a transcriptional level in LUAD. In addition, other studies have shown that the GSE101684 dataset from the Gene Expression Omnibus (GEO) database and discovered that circ-PLXND1, derived from the PLXND1 gene, was upregulated in NSCLC tissues. Functional deficiency tests have demonstrated that knocking down circ-PLXND1 could significantly inhibit the growth and metastasis of NSCLC cells and enhance cell apoptosis. Specifically, circ-PLXND1 silencing inhibits NSCLC progression via miR1287–5p upregulation and human Epidermal Growth Factor Receptor 3 (ERBB3) suppression (97) (**Figure 4B**). Moreover, Xia et al. (98) found that circ-0004140 was upregulated in LUAD (**Figure 4C**). Silencing circ-0004140 inhibited cell proliferation, migration, invasion, and tube formation, while inducing apoptosis in LUAD cells. These findings enhance our understanding of the regulatory functions of circRNAs in NSCLC and provide new insights into potential treatments for NSCLC.

#### 4.1.3 Epithelial-mesenchymal Transition

Under certain physiological or pathological situations, epithelial cells can transform into mesenchymal cells, resulting in cytoskeleton alterations, decreased adhesion, and loss of original cell polarity, which could improve cell motility and migration. This process could be summarized as EMT (99). Tumor metastases account for about 90% of cancer-related deaths (100, 101). According to research, EMT is a crucial process for epithelial cells to acquire mesenchymal properties and plays a crucial role in lung cancer cell metastasis and drug resistance (99, 102). Wang et al. (103) and Ge et al. systematically analyzed exosomes derived from NSCLC under hypoxic conditions and identified exosomal circPLEKHM1 as a hypoxia-induced circRNA that significantly promotes metastasis by inducing M2 macrophage polarization. *In vivo* experiments using the circPLEKHM1-specific reverse splicing sequence (circPLKHM1-ASO) showed a marked reduction in the expression of M2 macrophage markers (CD206 and CD115) and OSMR in tumors from mice treated with circPLEKHM1-ASO. Overall, circPLEKHM1 promotes NSCLC metastasis by regulating OSMR-mediated macrophage polarization. Consistently, Ge et al. (104) discovered that hsa-circ-0088036 silencing upregulated miR-1343–3p, inhibiting anti apoptotic protein 3 (Bcl-3) and NSCLC proliferation, invasion, and EMT. In this regard, hsa-circ-0088036 promotes NSCLC progression and metastasis and can function as an effective target for NSCLC treatment. (**Figure 4C**). In summary, these studies reveal a new circRNA-mediated mechanism and emphasize the significance of circRNA as a prognostic biomarker and therapeutic target for lung cancer metastasis.

#### 4.1.4 Metabolism

Besides the glycolytic metabolic pathway, circRNA also regulates NSCLC development through the glutamine metabolic pathway (105). Wang et al. (106) confirmed that circEPB41L2 induces polyubiquitination and degradation of PTBP1 by binding to its RRM1 structural domain and facilitating its interaction with the E3 ligase TRIP12. This interaction leads to the ubiquitin-mediated degradation of PTBP1, thereby blocking PTBP1-induced activation of the Pyruvate Kinase M2 subtype (PKM2). Consequently, this process inhibits glucose uptake, lactate production, and the progression and metastasis of NSCLC. Additionally, emerging evidence indicates that circRNAs play vital roles in tumor progression, including LUAD. Li et al. (107) identified a novel circRNA, circP4HB, derived from the P4HB gene, which promotes LUAD progression, accelerates aerobic glycolysis, and induces M2 macrophage polarization by interacting with PKM2. Furthermore, Glutamine, as a nitrogen source, is implicated in numerous synthetic metabolic processes in cancer. Zhu et al. (108) confirmed that circ-PDZD8 upregulates La Ribonucleoprotein 1 (LARP1) by targeting miR330–5p, thus promoting glutamine metabolism, which in turn enhances NSCLC proliferation and invasion. Taken together, these findings demonstrate that circRNAs can block NSCLC onset and progression by inhibiting metabolic pathways.

#### 4.1.5 Drug resistance

Chemoresistance is the leading cause of poor outcomes of NSCLC. Cisplatin (DDP) is the first-line anti-cancer chemotherapy drug for NSCLC treatment (109–111). In this regard, it is plausible that NSCLC progression correlates with DDP resistance. Research has indicated that circRNA increases cisplatin resistance in NSCLC. Specifically, circ-ANXA7 upregulates CCND1 (a cell cycle-related protein) by absorbing miR-545–3p, causing abnormal proliferation of NSCLC cells and accelerating their resistance to DDP (**Figure 4D**). However, other studies have confirmed (112) that circANKRD28 overexpression could significantly suppress tumor growth in mice while increasing DDP chemotherapy sensitivity. Furthermore, miR-221–3p has been reported to regulate NSCLC cell proliferation and migration, as well as cisplatin resistance, by targeting the Suppressor of Cytokine Signaling 3 (SOCS3). However, circANKRD28 bound to the miR-221–3p/SOCS3 axis, significantly inhibiting malignant biological progression and cisplatin resistance. These findings offer novel insights into addressing DDP resistance in NSCLC by targeting circRNA.

### 4.2 Nasopharyngeal cancer

Nasopharyngeal Carcinoma (NPC) is a malignant tumor originating from nasopharyngeal epithelial cells. Its occurrence is mostly linked to genetic factors, EB virus infection, and environmental factors (113). Furthermore, its incidence rate is extremely uneven, with obvious regional clustering and ethnic preferences, mostly in East and Southeast Asia, especially in southern China (114). As a cancer regulatory factor, circRNA modulates NPC onset and progression via mechanisms such as proliferation and migration, drug resistance, metabolism, and signaling pathways.

#### 4.2.1 Proliferation and migration

**Figure 5:**
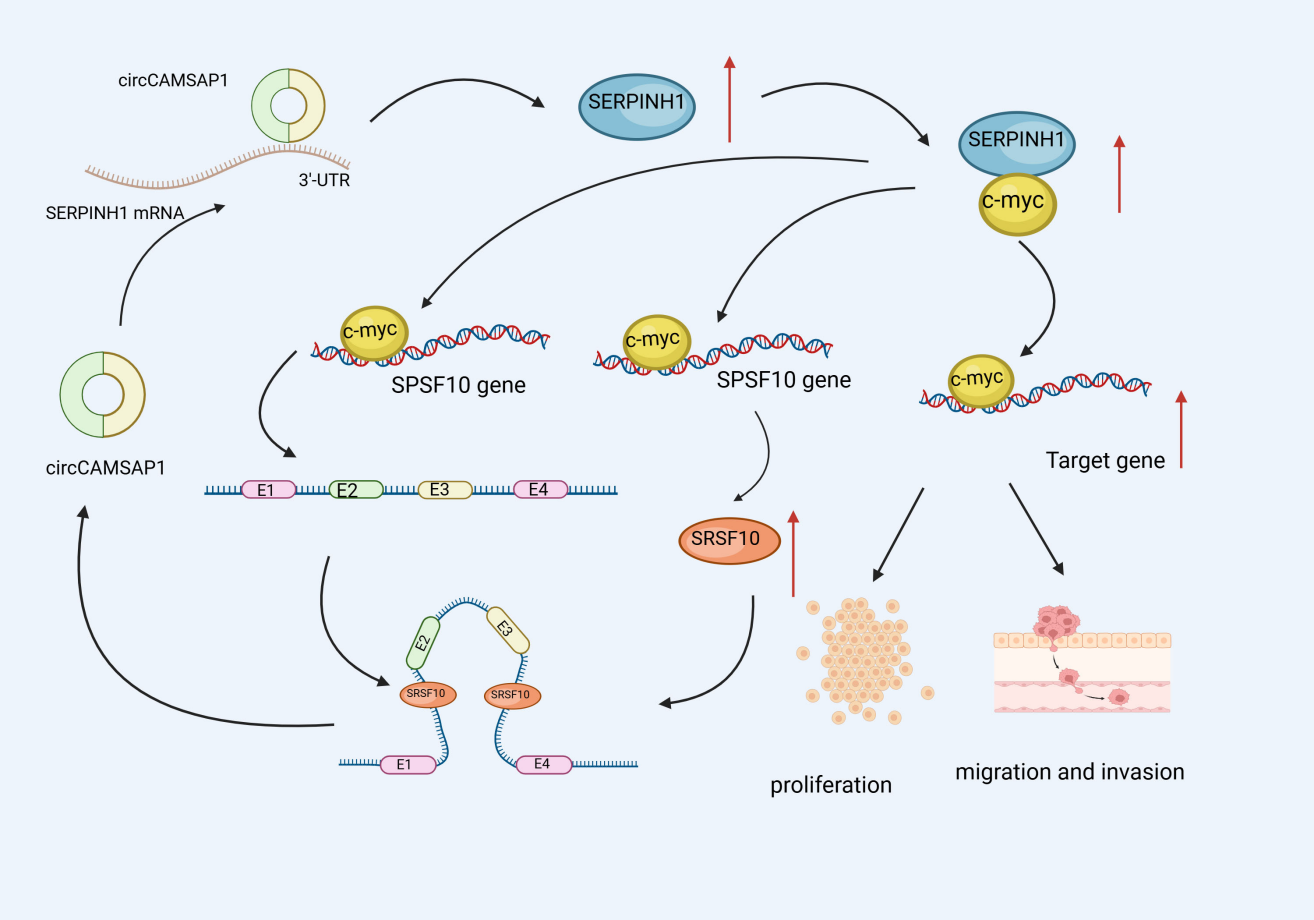
Schematic illustration of circCAMSAP1 promoting proliferation and metastasis of NPC through a positive SERPINH1/c-Myc feedback. circCAMSAP1 bound to and stabilized the 3’ untranslated region of the molecular chaperone SERPINH1 mRNA and promoted SERPINH1 expression.Additionally,the upregulated SERPINH1 protein bound to c-Myc, blocking the latter’s ubiquitination degradation. Furthermore, c-Myc stimulated SRSF10 expression and CAMSAP1 transcription, yielding pre-mRNA, whereas high SRSF10 expression stimulated pre-mRNA splicing, resulting in circCAMSAP1.Moreover C-Myc and SRSF10 jointly promoted circCAMSAP1 formation, resulting in the establishment of a positive feedback loop, leading to NPC proliferation and metastasis.

According to research, CircRNA is crucially involved in NPC metastasis and proliferation (115). As a major TF, C-Myc regulates over 15% of genes in the human transcriptome and can promote a range of cancer-related transcriptional processes associated with metastasis and proliferation (116, 117).Wang et al. (118)performed tests using cell and animal models and discovered that circCAMSAP1 stimulated NPC cell proliferation, invasion, and migration. Further investigations revealed that the upregulated SERPINH1 protein bound to c-Myc, blocking the latter’s ubiquitination degradation. Additionally, circCAMSAP1 bound to and stabilized the 3’ untranslated region of the molecular chaperone SERPINH1 mRNA and promoted SERPINH1 (a collagen synthesizing protein) expression. Furthermore, c-Myc stimulated SRSF10 expression and CAMSAP1 transcription, yielding pre-mRNA, whereas high SRSF10 expression stimulated pre-mRNA splicing, resulting in circCAMSAP1.Moreover C-Myc and SRSF10 jointly promoted circCAMSAP1 formation, resulting in the establishment of a positive feedback loop, leading to NPC proliferation and metastasis. These findings contribute to our understanding of the role circRNA plays in the pathogenesis of NPC (**Figure 5**).

#### 4.2.2 Drug resistance

Chemotherapy and radiotherapy are the primary forms of treatment for patients with advanced NPC (13). Paclitaxel is often used to treat NPC, and resistance to this medication contributes significantly to the unfavorable prognosis of NPC. Cheng et al. (69) confirmed the correlation between circRNA-0067717 and NPC paclitaxel resistance, reporting significant circRNA-0067717 upregulation in paclitaxel-resistant NPC cells. Specifically, the highly expressed circRNA-0067717 lowered the p53 protein levels by binding more p53 proteins and TRIM41 (an E3 ubiquitin ligase). This process promoted the TRIM41-mediated p53 ubiquitination and destruction. Given that it blocks cell cycle progression and induces apoptosis in potentially cancerous cells, P53 is essential for cancer growth prevention (119). Therefore, circRNA-0067717 increased the resistance of NPC cells to paclitaxel treatment by acting as a scaffold for TRIM41 and p53 and promoting TRIM41-mediated p53 degradation through ubiquitination.

#### 4.2.3 Metabolism

**Figure 6:**
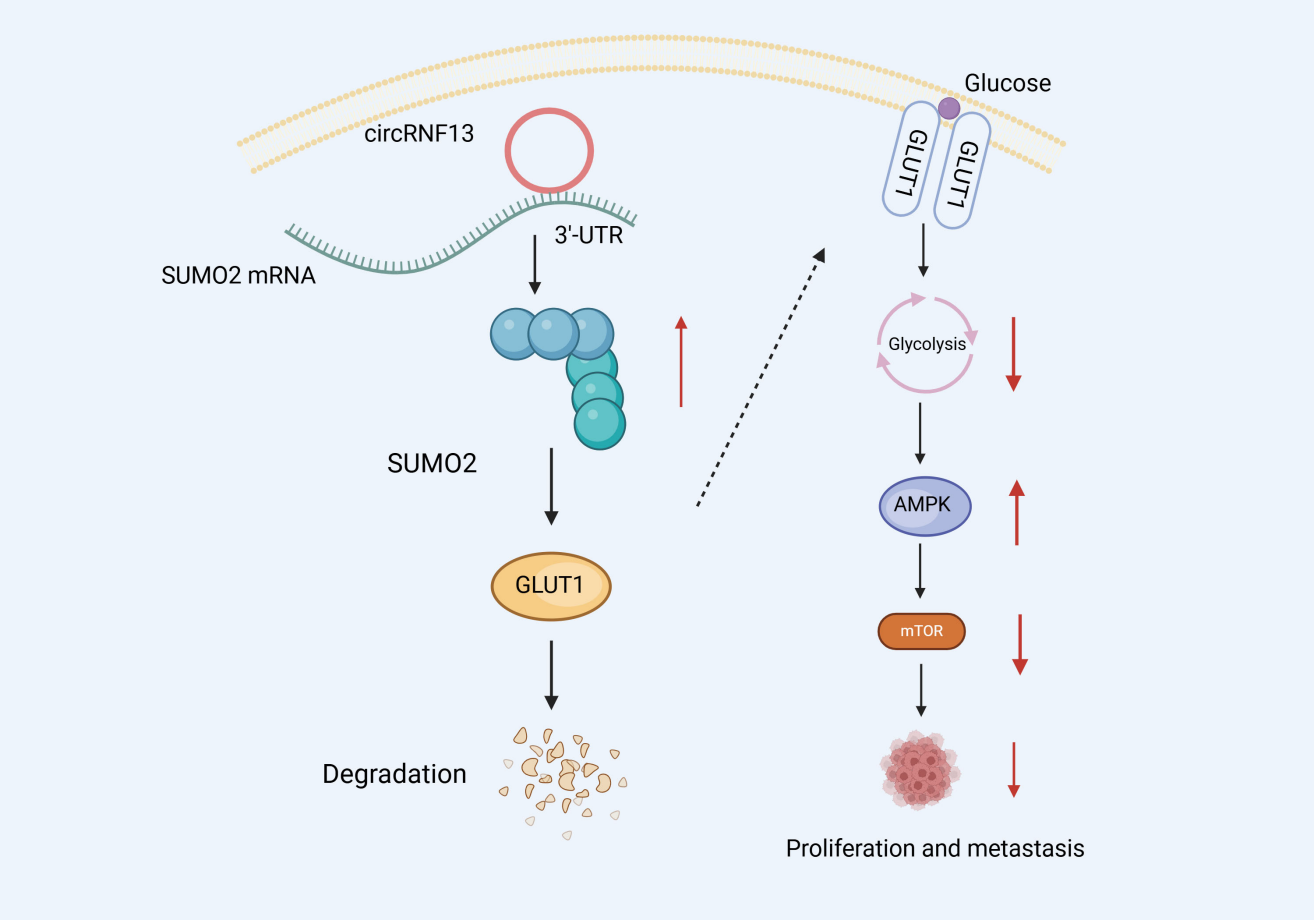
Schematic diagram of the molecular mechanism of circRNF13 in NPC.circRNF13 can directly bind to the 3 ‘- UTR region of Small Ubiquitin-like Modifier 2 (SUMO2), improving SUMO2 mRNA stability. SUMO2 overexpression could downregulate and promote the ubiquitination degradation of Glucose Transporter 1 (GLUT1), thus inhibiting the glycolysis of NPC cells. Glycolysis inhibition induces the activation and phosphorylation of the intracellular AMPK pathway. Subsequently, the activated AMPK pathway blocks protein synthesis and translation by suppressing the mTOR signaling pathway, thus inhibiting tumor growth and metastasis.

It has been established that circRNA inhibits NPC migration and invasion via glycolysis suppression. Mo et al. (114) performed RNA pull-down and dual luciferase experiments and confirmed that circRNF13 can directly bind to the 3 ‘- UTR region of Small Ubiquitin-like Modifier 2 (SUMO2), improving SUMO2 mRNA stability. Furthermore, Western Blot (WB) experiments revealed that SUMO2 overexpression could downregulate and promote the ubiquitination degradation of Glucose Transporter 1 (GLUT1), thus inhibiting the glycolysis of NPC cells. Glycolysis inhibition induces the activation and phosphorylation of the intracellular AMPK pathway. Subsequently, the activated AMPK pathway blocks protein synthesis and translation by suppressing the mTOR signaling pathway, thus inhibiting tumor growth and metastasis. These insights confirm that circRNF13 could exert tumor suppressive effects. By binding directly to SUMO2’s 3 ‘- UTR, circRNF13 could upregulate its expression, promoting GLUT1 ubiquitination degradation, which, in turn, may inhibit the glycolytic pathway and ultimately suppress NPC proliferation and migration (**Figure 6**).

#### 4.2.4 Signaling pathways

Signaling pathway activation regulates important cellular mechanisms such as cell proliferation, invasion, survival, inflammation, and immunity (120). For example, abnormal JAK/STAT signaling has been established to promote cancer progression and metastasis (121). Furthermore, through Reverse Transcription quantitative PCR (RT qPCR), Tian et al. (122) confirmed hsa_circle_0013561 upregulation in NPC, which is closely associated with the malignant progression of NPC. By activating the JAK2/STAT3 signaling pathway, hsa_cic_0013561 promotes NPC progression and accelerates cell cycle processes. Furthermore, IL-6, as an upstream activator of the JAK2/STAT3 pathway, could over-activate the JAK2/STAT3 signaling pathway, promote the malignant progression of NPC, and shorten patients’ survival time when upregulated, thus providing a potential target for NPC treatment.

### 4.3 Laryngeal squamous cell carcinoma

According to pathological classifications, squamous cell carcinoma accounts for 85%-96% of laryngeal cancer cases, making it one of the most common malignant tumors of the head and neck (123). Given its involvement in LSCC proliferation and invasion, circRNA has emerged as a viable target for LSCC treatment and detection (124). Despite a low incidence rate, LSCC has demonstrated a substantial mortality rate and < 50% overall 5-year survival rate (125). Consequently, exploring the mechanism of action of LSCC is imperative.

#### 4.3.1 Proliferation and migration

According to research, circRNA is closely associated with LSCC invasion and proliferation (126). Cyclin D1 (CCND1), a fundamental regulator of the cell division cycle, is one of the most often disrupted therapeutic targets in human cancer (127). Zang et al. (128) discovered that circ-CCND1 interacts with other molecules to prevent CCND1 mRNA decay, raising CCND1 expression after transcription, thus contributing to the development of LSCC tumors. Specifically, circ-CCND1 binds physically to the HuR protein, increasing CCND1 mRNA stability. On the other hand, circ-CCND1 could act as a sponge for miR-646, reducing its inhibitory effect on CCND1 mRNA. In summary, circ-CCND1 regulates interactions with HuR and miR-646, promoting cell proliferation, which, in turn, enhances CCND1 mRNA stability in LSCC, thus providing a theoretical basis for LSCC treatment using gene therapy.

#### 4.3.2 Metabolism

It has been established that Malic Enzyme 1 (ME1) can oxidize and decarboxylate malic acid to pyruvate, presenting a key cellular energy metabolism mechanism (129). Xu (130) (118) confirmed circSERPINA3 upregulation in LSCC using the qRT PCR dual luciferase assay and elucidated the circSERPINA3-miR-885–5 targeting relationship. Specifically, they discovered that circSERPINA3 could promote LSCC invasion and migration by regulating the miR-885–5p/ME1 axis. According to research, Orthodenticle Homeobox 1 (OTX1), a key regulatory factor for cancer development and progression, is a potential therapeutic target for tumors (131). Through microarray analysis, Li et al. (132) discovered high circMYOF expression in the plasma of LSCC patients. Furthermore, functional analysis revealed that circMYOF silencing inhibited glucose consumption and lactate production in LSCC cells. Moreover, bioinformatics analysis showed that circMYOF knockdown inhibited cell growth, metastasis, and glycolysis through the miR-145–5p/OTX1 pathway, slowing down the malignant processes of LSCC. Therefore, circMYOF holds great promise as an oncogenic gene in LSCC treatment.

## 5 Conclusion and outlook

In conclusion, circRNA, as a novel ncRNA, possesses highly conserved, stable, and specific biological characteristics and participates in various molecular mechanisms in cancer tissues and illnesses. With gradual advancements in bioinformatics technology and high-throughput sequencing methods, an increasing number of circRNAs have recently been identified in respiratory system cancers. Herein, we summarized the biogenesis, biological role, and molecular mechanisms of circRNA regulation in respiratory cancers. Although circRNA has the potential to be an effective biomarker for early cancer detection, diagnosis, and prognosis, research on its interaction with other molecular mechanisms, as well as the screening and analysis of functional circRNAs in respiratory system cancers, is still in its early stages, necessitating more clinical and biological investigations. Furthermore, the interaction between circRNAs and other molecules should be validated through omics research. Future explorations in the aforementioned research areas will contribute to the clinical diagnosis and treatment of cancer.

## Author contributions

NY: Writing – original draft. MJ: Writing – original draft. YZ: Writing – review & editing. SM: Writing – review & editing. LW: Visualization, Writing – review & editing. JL: Funding acquisition, Writing – review & editing.
